# Depressive primary care patients’ assessment of received collaborative care

**DOI:** 10.1038/s41598-023-29339-9

**Published:** 2023-02-09

**Authors:** K. Lukaschek, C. Beltz, S. Rospleszcz, H. Schillok, P. Falkai, J. Margraf, J. Gensichen

**Affiliations:** 1 Institute of General Practice and Family Medicine, University Hospital, LMU Munich, Nussbaumstraße 5, St.-Vinzenz-Haus, 80336 Munich, Germany; 2 Department of Epidemiology, Institute for Medical Information Processing, Biometry and Epidemiology, Ludwig-Maximilians-Universität München, Munich, Germany; 3 Institute of Epidemiology, Helmholtz Zentrum München, German Research Center for Environmental Health, Neuherberg, Germany; 4 Graduate Programme “POKAL - Predictors and Outcomes in Primary Care Depression Care” (DFG-GrK 2621), Munich, Germany; 5 Department of Psychiatry and Psychotherapy, University Hospital, LMU Munich, Munich, Germany; 6 Mental Health Research and Treatment Center, Ruhr-University Bochum, Bochum, Germany

**Keywords:** Health care, Medical research

## Abstract

The “Patient Assessment of Chronic Illness Care” (PACIC) is a tool for evaluating outpatient health service for patients with chronic diseases. Our aim was to analyze the association between PACIC scores of primary care patients with depression and patients’ or patients’ general practitioners’ (GPs) characteristics. In a data set including depressive primary care patients (N = 280) the association of patient characteristics (sex, age, depressive symptom severity, suicidal ideation) with PACIC scores were assessed by linear regression models. The association between GPs’ characteristics (type, location of practice; age, qualification of practitioner) and PACIC scores was assessed by linear mixed models with individual practices as random effects. Patient Health Questionnaire (PHQ-9) scores at 12 months follow up and changes in PHQ-9 scores from baseline to follow up were significantly positive associated with higher PACIC scores (beta = 0.67, 95%-CI [0.02, 1.34]). PACIC scores were not associated with patients’ sex (p = 0.473) or age (p = 0.531). GP’s age was negatively associated with PACIC scores (p = 0.03). In conclusion, in patients with depression, the PACIC is independent from patients’ and GPs’ characteristics. The PACIC may be appropriate to assess patient-perspective on depression services in primary care.

## Introduction

Depression, a heterogeneous condition with a chronic and recurrent course, is frequently seen in primary care setting. Collaborative delivery models, such as the chronic care model (CCM), are a cost-effective strategy to deliver depression interventions in routine care^1^ For the general practitioner (GP), it can be challenging to recognize and treat depression, particularly in patients with multiple comorbidities^2^ Thus, including the patients’ perspective on health delivery has emerged as an approach to aid primary care providers in improving care of patients with depression^3^

CCM is an evidence-based guide to collaborative and chronic care improvement that enables the management of several chronic diseases such as depression^4,5^ CCM suggests that better medical outcomes for chronic disease occur when there is coordinated, patient-centred care and a productive interaction between an active, informed patient and a proactive practice team^4,6,7^. The combination of four principles promotes this interaction: self-care (self-management, behavioural activation), coordination (teamwork, case management), decision-making support (evidence-based guidelines), IT/data (clinical patient, practice and routine data)^8^:

- *Patient self-management*, or the active involvement of patients in the treatment process, strengthens the patient’s role. Among other things, it involves assisting in making treatment decisions, keeping an eye on clinical results, generating personal health data, and dealing with special disease situations safely.
- *Coordination* of all participants, and interdisciplinary task sharing, for example within practice teams, i.e. family doctors, medical assistants and patients, but also between family doctors, patients and psychiatrists/psychotherapists. The aim of coordination is to improve clinical care processes and treatment, e.g. through monitoring (case management).
- *Decision-making support* Clinical diagnostic and therapeutic decisions are science-based, and take into account, for example, evidence-based guidelines for physicians and patients.
- *IT and data collection* ensure quality assurance in clinical care. Patient records and registers, as well as routine and patient-generated health data, are used to plan the treatment of individual patients and entire patient groups, e.g. in family practices.

The Patient Assessment of Chronic Illness Care (PACIC) was developed to document patients’ perspective of the extent to which their treatment and care of the last 6 months is in accordance with the CCM^9^. Thus, the PACIC can be a powerful tool for quality assurance and quality improvement of chronic care, contributing to reducing the gap between research findings and practical delivery, which is known to exist especially for depression^3^. The PACIC not only documents the depressive patients’ perspective on their received care, but also balances out potentially biased perception of care providers regarding their services and create a more holistic picture of chronic care delivery for depressive patients^9^. The original PACIC included 20 items, but now there are several shorter versions. The PACIC is designed around five subscales: (a) patient activation, (b) delivery system design and decision support, (c) goal setting and tailoring, (d) problem-solving and contextual counselling, (e) follow-up and coordination. Higher PACIC scores indicate better perceived alignment of the received care with the CCM^9^. Only view studies examine PACIC-scores in a population of primary care patients with depression: Glasgow et al. included 255 patients having 1 or more chronic illness in their study, among those 51 with depression, but these patients did not statistically differ regarding their PACIC scores from patients with other chronic illnesses (e.g. Asthma, Diabetes, Hypertension, Arthritis)^10^. They also reported correlations of the PACIC and its subscales with patient characteristics (all ≤ 0.25); higher overall PACIC-scores were related to higher age higher and female sex, the latter also significantly associated with all subscales (0.14 to 0.25; p < 0.05). To our knowledge, the only study specifically investigating the PACIC among patients with depression is Gensichen et al. who evaluated the psychometric properties of the German 20-item PACIC version in a sample of patients with major depression (n = 442)^11^. This cross-sectional study was nested in the PROMPT trial on the effectiveness of case management. Overall, the mean PACIC score among patient with major depression was 3.25, and there were no associations of patients’ characteristics (e.g. sex, age) with PACIC scores.

The aims of the study were to analyse whether there are association between PACIC scores of primary care patients with depression who received case management and patients’ characteristics (sex, age, severity of depression, suicidal ideation) or patients’ general practitioners’ (GPs) characteristics (type of practice, location of practice, age of GP, and qualification in “primary psychosomatic care”). Patients’ characteristics can influence the patients’ perception and reporting of care and thus, their PACIC assessment; GPs’ characteristics could influence the actual care received. Regarding a patient-centred care delivery, understanding what drives patient satisfaction with their received care and which covariates and context factors might affect their perception is a key aspect to improve high-quality patient care.

## Methods

### Study design and setting

A pooled data analysis was performed, including two RCTs on two different psychiatric disorders, panic disorder and depression. In both RCTs, patients in the intervention group received case management by a medical assistant, patients in the control group received treatment as usual. The PARADIES (Patient Activation foR Anxiety DIsordErS) trial enrolled patients with panic disorder with/without agoraphobia (ICD-10: F41.0 or F40.01) to a two-armed cluster-randomised intervention study (brief cognitive behavioural therapy-oriented intervention group versus treatment-as-usual control group)^12^. The intervention group (n = 230 patients from 36 practices) received a patient guidebook and four doctor appointments within 23-weeks. A practice-based medical assistant (MA) carried out telephone monitoring at regular intervals. The control group (189 patients from 37 practices) received routine treatment. The PACIC S-11 was used to capture the patient’s view of their treatment.

The PROMPT (PRimary care Monitoring for depressive Patients Trial) trial enrolled patients with major depression (ICD-10: F32.2) to a two-armed cluster-randomised intervention study^13^. In the intervention group (n = 310 patients from 35 practices), patients received case management in accordance with the CCM, including structured telephone interviews conducted by MAs at regular intervals to monitor depression symptoms, and support for adherence to medication and/or behavioural activation. The control group was treated according to routine care. The 20-item version of the PACIC was used to capture the patient’s view of their treatment.

For both studies, patient characteristics and depressive symptoms (PHQ-9) were recorded at baseline (T0), after 6 month of follow-up (T1) and after one year of follow-up (T2). PACIC scores were recorded at one year of follow up (T2). The rationale behind the interventions—a team-based approach to and case-management of a mental disorder—was comparable in both trials.

The studies were approved by the Ethics committees of the Friedrich-Schiller University Jena (no. 3484–06/1) and the University of Frankfurt (no. E 26/05), respectively, and complied with the Declaration of Helsinki including written informed consent from all patients. They were both registered at Current Controlled Trials (ISRCTN64669297; ISRCTN66386086).

### Primary outcome: the PACIC score

The PACIC is a self-report tool for patients and reflects the patient’s perspective on the care of their chronic health conditions. It asks to what extent the treatment was in accordance with the CCM using five subscales (Patient Activation/Involvement, Delivery System Design/Decision Support, Goal Setting/Tailoring, Problem Solving/Contextual, Follow-up/Coordination)^9^. Higher scores are indicative of the perception of better care and can thus be viewed as a surrogate measures of patient satisfaction.

In PRoMPT, the 20-item version of the PACIC was used with response options according to a Likert scale with five scale levels (“almost never” to “almost always”)^9^. In PARADIES, a 11-item PACIC version using a 11-point percentage scale from 0 to 100% (with 10%-intervals, 0% = “none”, 100% = “always”) was applied^14,15^.

To our knowledge, there are no studies pooling data from studies using the PACIC 20-item version and the PACIC 11-item version. However, Arditi et al. developed a strategy to compare the overall score of the PACIC 11-item, PACIC 20-item, and the PACIC-5As 26-item versions by matching the 11-point response scale of the short PACIC version with the 5-point Likert scale of the longer versions^16^. In the present study, we had to compare the five-point Likert scale with the eleven-point percentage scale; therefore, we calculated a new measure “percentage of maximum” for each participant, which was standardized per study by design. First, we excluded all items from the 20-item version which are not present in the 11-item version. Second, participant’s answers were summed up and divided by the maximum attainable score of the respective score version. An example is shown in the Supplementary Text S2. Multiplied with 100, this results in a standardized percentage value, which can be compared between the two score versions, ranging from 0 (low patient satisfaction) to 100 (high patient satisfaction). In this analysis “PACIC score” always refers to this newly calculated “percentage of maximum”.

### Measurement of depressive symptoms

The Patient Health Questionnaire 9 (PHQ-9) is the depression module of the Patient Health Questionnaire (PHQ) and consists of the 9 criteria on which the diagnosis of depressive disorders is based^17^. With reference to the last 2 weeks, the patients indicate with the help of a Likert scale whether the diagnostic criteria “Not at all”, “Several days”, “More than half the days” or “Nearly every day” apply. By coding the individual scale levels with the values 0–3, the overall score is formed from the sum (0–27 points) of all answers. The higher the overall score, the higher the severity of depression. The last item “Thoughts that you would be better off dead or hurting yourself in some way” is considered an indicator for suicidal ideation if “Several days” ore more often is answered. The PHQ-9 is applicable if a maximum of 2 of the 9 items were not answered. In the case of an incomplete questionnaire, the missing values were imputed by means of the own point average (valid mean substitution, VMS).

### Patient inclusion criteria

**Figure 1:**
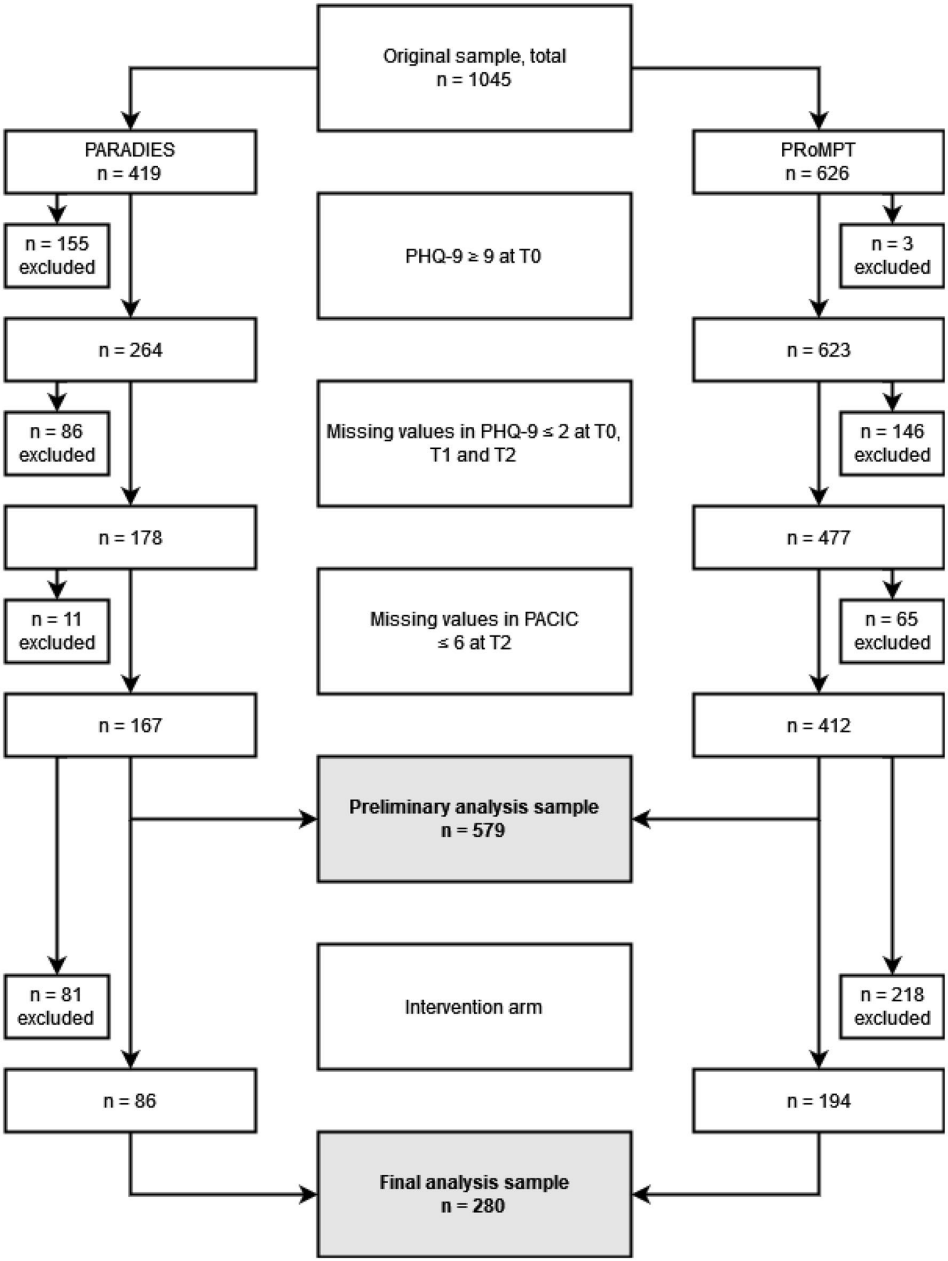
Flowchart.

Patient flow for this analysis is depicted in Fig. 1. Briefly, participants were excluded if they did not have depression at baseline (PHQ-9 ≤ 9), or were excluded due to missing values in either the PHQ-9 score or the PACIC score. Furthermore, after comparing PACIC values across intervention and control arm of each study in a preliminary analysis, only the intervention arms were included in the final subsequent analysis.

### Statistical analysis

Patient and practice characteristics (see Table 1) are presented as medians with first and third quartile and arithmetic mean with standard deviation for continuous variables and counts and percentages for categorical variables. Differences in these characteristics according to study were evaluated by Mann-Whitney U test or Χ^2^ test, respectively.

**Table 1:** Patients’ and GPs’/practice characteristics (intervention arms).

		Pooled sample	PARADIES	PRoMPT	p-value
		N = 280	N = 86 (30.7%)	N = 194 (69.3%)	
Patient characteristics					
Age, years	Median	51.0 [41.8, 58.0]	47.0 [36.2, 53.0]	53.0 [43.0, 61.8]	0.002
	Mean	50.2 ± 13.6	46.3 ± 11.8	52.0 ± 14.0	< 0.001
Men		71 (25.4%)	23 (26.7%)	48 (24.7%)	0.837
PHQ-9 Score at T0, points	Median	16.0 [13.0, 19.0]	14.0 [11.0, 17.0]	17.0 [14.0, 19.0]	< 0.001
	Mean	16.3 ± 3.9	14.5 ± 4.2	17.0 ± 3.6	< 0.001
PHQ-9 Score at T1, points	Median	11.0 [6.0, 15.0]	9.0 [5.0, 12.0]	12.0 [7.0, 16.0]	< 0.001
	Mean	11.0 ± 5.6	9.0 ± 4.9	11.9 ± 5.6	< 0.001
PHQ-9 Score at T2, points	Median	9.0 [6.0, 14.0]	8.0 [4.0, 12.0]	10.0 [6.2, 14.0]	< 0.001
	Mean	9.9 ± 5.8	8.1 ± 5.2	10.7 ± 5.9	< 0.001
Change PHQ-9 T2–T0, points	Median	− 6.6 [− 10.0, − 3.0]	− 6.1 [− 9.0, − 2.0]	− 7.0 [− 10.0, − 3.0]	0.636
	Mean	− 6.3 ± 5.3	− 6.3 ± 5.3	− 6.3 ± 5.3	0.614
Depression at T2 (PHQ-9 ≥ 9)		148 (52.9%)	32 (37.2%)	116 (59.8%)	0.001
Potential suicidal ideation at T2		83 (29.6%)	16 (18.6%)	67 (34.5%)	0.011
PACIC, % of maximum at T2	Median	69.1 [54.5, 83.6]	71.8 [43.2, 83.6]	69.1 [56.4, 83.6]	0.519
	Mean	67.8 ± 20.0	64.7 ± 25.0	69.2 ± 17.2	0.314
Practice characteristics					
Based on n = 242 patients					
Type of practice					0.002
Single		151 (62.4%)	42 (48.8%)	109 (69.9%)	
Group		91 (37.6%)	44 (51.2%)	47 (30.1%)	
Location of practice					< 0.001
Urban		160 (66.1%)	22 (25.6%)	138 (88.5%)	
Rural		82 (33.9%)	64 (74.4%)	18 (11.5%)	
GP qualified for “basic psychosomatic care”		68 (28.1%)	58 (67.4%)	10 (6.4%)	< 0.001
Year practice founded^#^	Median	1997.0 [1987.0, 2001.0]	1999.0 [1993.8, 2006.0]	1989.0 [1985.5, 2000.0]	< 0.001
Practice founded after study specific median^#^		130 (53.7%)	50 (58.1%)	80 (51.3%)	0.277
Age of study physician, years^#^	Median	49.0 [44.0, 56.0]	51.0 [47.0, 54.5]	49.0 [43.0, 56.0]	0.387
	Mean	50.5 ± 7.3	50.9 ± 6.9	50.3 ± 7.5	0.572

Continuous variables are presented as median [1st quartile, 3rd quartile] with p-values from Mann-Whitney U test and mean ± standard deviation with p-values from t-test. Categorical variables are presented as counts and percentages with p-values from Χ^2^-Test.

^#^Based on n = 240.

Differences in PACIC scores according to categorical characteristics were displayed graphically by boxplots and quantified by Mann-Whitney U test. The age of a practice was categorized into “new” or “old” according to the study specific median foundation year. Correlations between PACIC scores and continuous variables were displayed by scatterplots and evaluated by Pearson’s correlation coefficient with corresponding 95%-Confidence Intervals (CI). As a sensitivity analysis, single items of the PACIC score were exploratively compared according to depression status. To account for potential confounders and assess effect sizes beyond correlations, linear mixed regression models with random intercepts per practice and PACIC scores serving as outcome were calculated to obtain beta coefficients and corresponding 95%-CI as measures of association for patient and practice characteristics. We chose random effects per practice, since characteristics of patients visiting a certain practice might be similar, thus conferring a clustering structure in the data. Obtained beta coefficients represent changes in patients’ PACIC score per one unit or category change in the variable of interest.

SPSS v26 and R v4.0.3 were used for all calculations. p-values < 0.05 are considered to denote statistical significance. In this exploratory analysis, no additional correction for multiple testing was performed.

## Results

The final analysis sample included N = 280 depressed patients from 62 practices, who had received the intervention in their respective study. Patients’ and GPs’ characteristics are presented in Table 1 and Supplementary Table 1.

### Association of PACIC scores and treatment

PACIC scores were significantly different between the control and intervention arm within both studies. Median PACIC values for the PARADIES study were 44% and 72% (p < 0.001) and for the PRoMPT study 62% and 70% (p < 0.001) for control and intervention group, respectively. There was no significant difference in PACIC values between the intervention arms of the two studies (p = 0.519).

In a linear mixed model adjusted for patient age, sex and suicidal ideation at time T2 with random intercept per practice, higher PHQ-9 scores at T2 and higher improvement in PHQ-9 scores from baseline to T2 were significantly associated with higher PACIC scores (beta = 0.67, 95%-CI [0.02, 1.34]).

In a sub analysis, we tested for each of the included 11 PACIC-items whether the item differed between the group with depression (PHQ-9 ≥ 9) and the group without depression. There were nominally significant differences for two items from the PACIC subscale “Goal setting” (“I was given a copy of my treatment plan”, p-value: 0.027; “I was encouraged to go to a specific group or class to help me cope with my chronic condition”, 0.025) and for two items from the PACIC subscale “problem solving/contextual counselling” (“I was helped to make a treatment plan that I could carry out in my daily life”, 0.019; “I was helped to plan ahead so I could take care of my condition even in hard times”, 0.012). We note, however, that these differences would not be statistically significant anymore after correction for multiple testing.

### Association of PACIC scores and patient characteristics

There were no significant differences between male and female participants (p = 0.473) or between subjects suicidal and non-suicidal according to the PHQ-9 item 9 at T2 (p = 0.751). Furthermore, patient age was not significantly correlated with PACIC scores (r = 0.04, 95%-CI [− 0.07, 0.15], p = 0.531). As stated above, in a linear mixed model including patient age, sex, and suicidal ideation with random intercept per practice, none of these were significantly associated with PACIC scores (age: beta = 0.04, 95%-CI [− 0.14, 0.22]; female sex: beta = 1.16, 95%-CI [− 4.11, 6.47]; suicidal ideation: beta = − 1.30, 95%-CI [− 7.07, 4.46]).

### Association of PACIC scores and GP/practice characteristics

**Figure 2:**
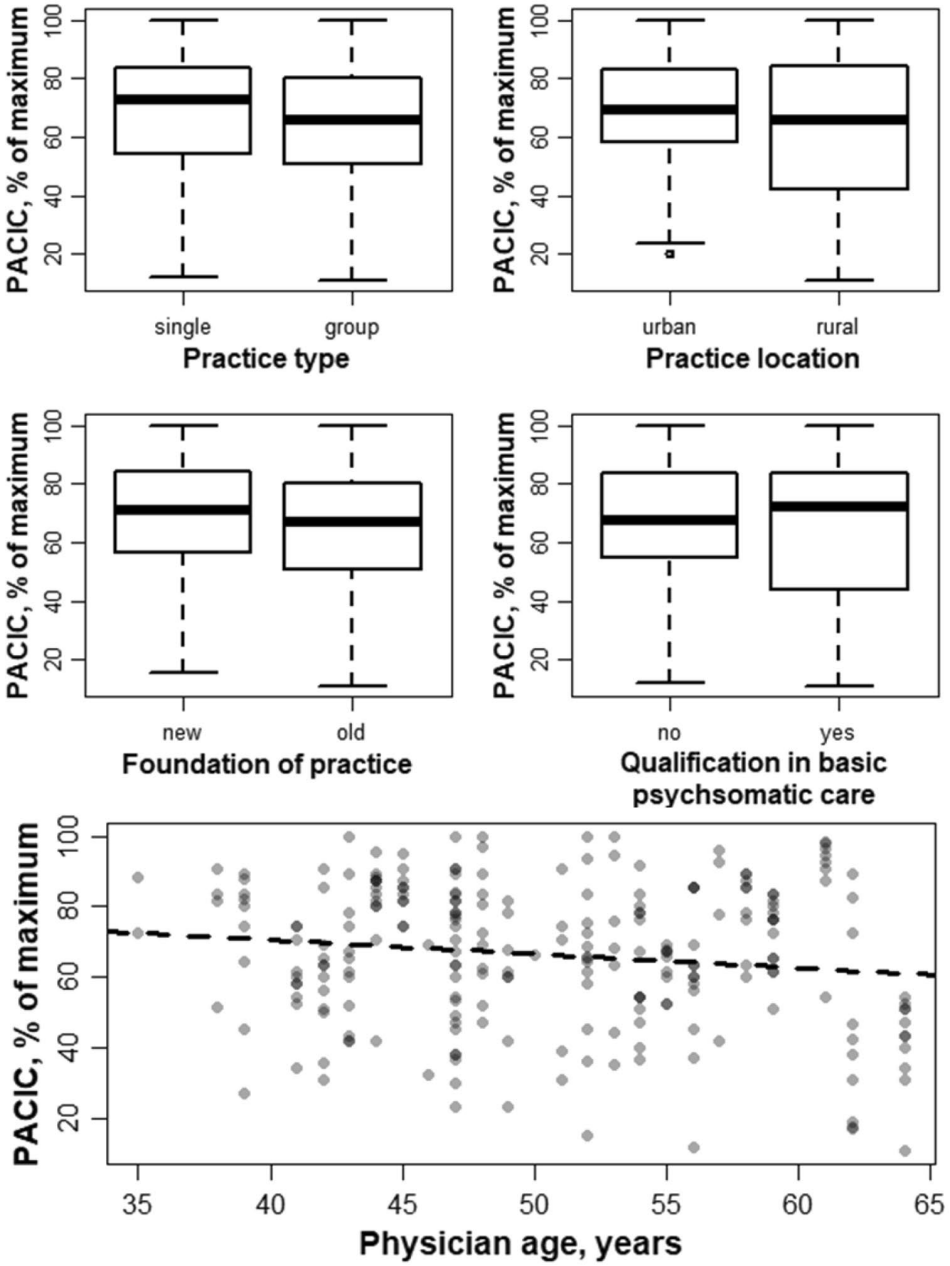
PACIC scores according to GP/practice characteristics. PACIC Scores are depicted on the y-axis, GPs’ characteristics (practice type, location, founding year; GPs qualification)on the x-axis of each box plot (upper two rows). The scatter plot on the bottom depicts the correlation of PACIC scores (y-axis) and GP’s age in years (x-axis) which was significantly negatively correlated.

For practice characteristics, there were no significant differences in PACIC scores between treatment in a single practice or in a group practice (p = 0.129), between urban and rural practices (p = 0.203), between practices founded before or after the study-specific median (p = 0.143), and between physicians with or without the additional qualification “basic psychosomatic care” (p = 0.785). Nevertheless, there were nominal differences in PACIC values according to these characteristics (see Fig. 2). This could be explained by the age of the treating physician: physician’s age was significantly negatively correlated with PACIC scores (r =  − 0.14, 95%-CI [− 0.27, − 0.02], p = 0.03, see Fig. 2). Physicians in urban practices were significantly younger compared to physicians in rural practices (median 47 years vs 53 years, respectively, p < 0.001) and physicians in more recently founded practices were significantly younger compared to physicians in older practices (median 45 years vs 56 years, respectively, p < 0.001). There were no significant age differences between physicians in single and group practices, between physicians in the two studies, or between physicians with and without the additional qualification “basic psychosomatic care”.

In a mixed-effects regression model taking into account the cluster structure given by the GPs’ practices, higher age of the treating physician was significantly associated with lower PACIC values (beta =  − 0.62, 95%-CI [− 1.18, − 0.07]; corresponding to a decrease of 0.62 percentage points in PACIC with a 1 year increase in treating physician’s age, compare Fig. 2). The association persisted after adjustment for practice type and presence of the additional qualification “basic psychosomatic care”, but attenuated after adjustment for practice location and time of foundation.

## Discussion

In this sample combining data from two randomized trials including primary care patients with depression we analysed potential determinants of PACIC scores as a measure of patients’ perspective. Our findings are: Depressive patients of collaborative interventions showed significantly higher PACIC scores than controls. Patients’ sex and age were not associated with PACIC scores. Practice characteristics, such as practice location and year of foundation, were associated with PACIC scores via the age of the GP. Younger GPs’ age was significantly associated with higher PACIC scores.

While Glasgow et al.^9^ found a slight correlation of the PACIC score with sex and age, we found no difference in the PACIC score between male and female subjects and between younger and older subjects. Our results are consistent with other research, were sex and age of the patient had no association with PACIC scores^18^. Thus, the PACIC seems to be mostly independent on socio-demographic factors, especially for depressive patients.

Rosemann et al.^19^ found in their analysis with the PACIC 5-A that younger and less depressed subjects achieved higher PACIC scores. In our analysis, higher PHQ-9 scores at T2 were significantly associated with higher PACIC scores. In a sub analysis comparing single PACIC items between the group with depression and the group without depression, there were nominally significant differences in items from the PACIC subscales “Goal setting” and “problem solving/contextual counselling”. Goal setting is a key element of cognitive behavioural therapy^20^ which was the underlying theoretical principle of both the PROMPT and the PARADIES study. Thus, GPs might have unconsciously focused on this issue.

In our analysis, there was no difference in the PACIC scores between subjects who were considered non-suicidal or possibly suicidal according to the last item of the PHQ-9. This could be due to the fact that especially the trust in the doctor and the feeling of being in good hands with the doctor is not explicitly asked by the PACIC.

There were no differences in the PACIC score with regard to the type of practice (single practice or group practice) and the location of the practice (in the city or in the country). This was probably due to the fact that we analysed patients in the intervention groups: their treatment was precisely predetermined by the study protocol and less dependent on the type of practice and the location of the practice. The fact that there were no differences between older and newer practices also supports the assumption that factors of the practices play less of a role due to the regulated process of the studies and are not explicitly queried by the PACIC.

Our analysis did not show any differences in the PACIC scores regarding the question of whether the GP had the additional qualification “basic psychosomatic care” or not. However, GPs in the intervention were specially trained for the intervention and were thus confident in treating mental disorders. However, the PACIC may not adequately address the mental health needs of patients, as the PACIC refers to the CCM and the CCM generally aims to improve outpatient care for chronic patients in general.

In our study, PACIC scores of patients of younger GPs were higher than the scores of patients of older GPs. We assume that younger GPs have not yet adopted a “professional role” and are therefore more likely to follow the guidelines of a study setting^21,22^ and are more enthusiastic when implementing new skills in everyday practice^23^. Due to the increasing complexity of everyday medical life with new challenges such as telemedicine, the increased importance of dealing with patients at eye level in medical studies and the omnipresent time pressure, younger GPs in particular may place a greater focus on planned procedures and tight schedules—points of organisation that are partly queried by the PACIC.

Another reason why patients of younger GPs had higher PACIC scores could be the growing importance of shared decision making (SDM) in medical education^24–27^. SDM encourages care teams and patients to discuss reasonable healthcare options together, using the best evidence available to support patients in considering options and achieving informed preferences^28^. Thus, GPs who have already encountered SDM during their training might have earned better PACIC scores^29^.

## Limitations

The strength of this analysis is the use of data from two well-designed studies which accounts for data-quality as well as quantity. As a limitation, we focused on the improvement of depression scores in both studies, although the primary outcome in one study was panic disorder. However, the association between the PACIC-score and depression severity were independent of the intervention, indicating that the PACIC is a robust measure of patients’ perspective on the care of their chronic health conditions. The presented method for comparing PACIC scores of different scale levels was feasible; however, the literature describes that e.g. the number of points on the response scale could affect the PACIC score in diabetic patients. On a broader level, it was shown that these elements of response style influence the answering behaviour of individuals on Likert scales^30^. It yet remains unclear if this pattern applies to depression and mental conditions as well. Therefore, when interpreting pooled results of different PACIC scale levels, one should carefully consider potential biases of this comparative method in terms of validity and comparability.

## Conclusions

The PACIC is a patient-level assessment of implementation of the CCM and suitable instrument to assess chronic care services. Since the PACIC in depressive patients is largely independent of patient and GP/practice characteristics, it may be a relevant tool in assessing and improving the quality of chronic disease care of primary care patients with depression.

## Supplementary Information

Supplementary Information 1.

Supplementary Information 2.

## Data Availability

The data that support the findings of this study are available from the consortia of the PARADIES and PROMPT studies, but restrictions apply to the availability of these data, which were used under license for the current study, and so are not publicly available. Data are however available upon reasonable request. Data requests may be directed at “Stiftung Allgemeinmedizin—The Primary Health care Foundation” (www.stiftung-allgemeinmedizin.de). Mail: office@stiftung-allgemeinmedizin.de.
