# Supplementary material for: Depressive primary care patients’ assessment of received collaborative care
Source: Sci Rep. 2023 Feb 9;13:2329. doi: 10.1038/s41598-023-29339-9 (PMC9911390; doi:10.1038/s41598-023-29339-9)
Supplement: Supplementary file 1 — Supplementary Information 1. [file 41598_2023_29339_MOESM1_ESM.docx]

eSupplement: methodological explanations

*The psychometric properties of the different versions of the PACIC*

In PRoMPT, the 20-item version of the PACIC was used with response options according to a Likert scale with five scale levels (“almost never” to “almost always”) (Glasgow et al., 2005). This version showed overall good psychometric properties with an excellent Cronbach’s α coefficient for the total scale of 0.91 indicating internal consistency. Construct validity was assessed via a Spearman rank correlation with the European Task Force on Patient Evaluations of General Practice (EUROPEP) and proved to be significant for all subscale (P <.05), ranging from -0.17 to -0.53. Possible ceiling effects für subscales seemed to be higher (8.9-12.9%) compared to possible floor effects (4.6 %).

In PARADIES, a 11-item PACIC version using a 11-point percentage scale from 0% to 100% (with 10%-intervals, 0% =”none”, 100% =”always” ) was applied (Goetz et al. 2012; Gugiu et al, 2009). This version has also reportedly good psychometric properties. Internal consistency was found to be 0.87 (Cronbach’s α). Validity was assessed as a Spearman rank correlation for the overall score with the 20-item PACIC and resulted into 0.82 (P <.001). The items demonstrated skewness levels close to zero and a factor analysis underlined the one-dimensional structure of the instrument.

- Glasgow, R. E. et al. (2005) Development and validation of the Patient Assessment of Chronic Illness Care (PACIC). *Med Care* **43**, 436-444, doi:10.1097/01.mlr.0000160375.47920.8c
- Goetz, K. et al. (2012) Adaptation and psychometric properties of the PACIC short form. *Am J Manag Care* **18**, e55-60.
- Gugiu, P. C. et al. (2009) Development and evaluation of the short version of the Patient Assessment of Chronic Illness Care instrument. *Chronic Illn* **5**, 268-276, doi:10.1177/1742395309348072.

*PHQ-9 and PACIC: Dealing with missing values*

In previous studies on the PHQ-9 (e.g. Arrieta et al. 2017; Kocalevent et al. 2013; Lowe et al. 2008), if the missing value was less than 20%, the missing value was replaced with the average of the remaining items. If the number of items missing from the scale exceeded 20% (2 items) of the total number of items, they were not counted in the total score and were treated as missing data. This procedure is recommended by the original authors (Spitzer et al. 1999; Kroenke et al. 2010). For our analysis, this meant that a total of 11 patients had imputed values on one PHQ-9 item across all three time points (T1: n=5 patients; T2: n=4; T3: n=2).

- Arrieta, J., et al. (2017). Validity and utility of the Patient Health Questionnaire (PHQ)‐2 and PHQ‐9 for screening and diagnosis of depression in rural Chiapas, Mexico: A cross‐sectional study. Journal of clinical psychology, 73(9), 1076-1090.
- Kocalevent R., et al. (2013) Standardization of the depression screener patient health questionnaire (PHQ-9) in the general population. Gen Hosp Psychiatry 35:551–5.
- Kroenke, K., et al. (2010). The Patient Health Questionnaire somatic, anxiety, and depressive symptom scales: A systematic review. General Hospital Psychiatry, 32(4), 345– 359.
- Lowe B., et al. (2008) Depression, anxiety and somatization in primary care: syndrome overlap and functional impairment. Gen Hosp Psychiatry 30:191–9.
- Spitzer, R. L., Kroenke, K., & Williams, J. B. (1999). Validation and utility of a self-report version of PRIME-MD: The PHQ primary care study. Primary Care Evaluation of Mental Disorders. Patient Health Questionnaire. JAMA: The Journal of the American Medical Association, 282(18), 1737– 1744.

Regarding the PACIC, we followed recommendations by Goetz et al. (2012) and Gugiu et al. (2009) stating that the PACIC-11 works with a mean value over the items and has a high internal consistency, and thus, is relatively insensitive to individual missing items. Questionnaires with more than half of the items missing were thus excluded from the analysis. This procedure has already been done in the original PARADIES study (Gensichen, 2019).

- Gensichen, J. et al. (2019). Panic Disorder in Primary Care: The Effects of a Team-Based Intervention—a Cluster-Randomized Trial. *Deutsches Aerzteblatt International*, *116*(10), 159.
- Goetz, K. *et al.* (2012) Adaptation and psychometric properties of the PACIC short form. *Am J Manag Care* **18**, e55-60.
- Gugiu, P. C. et al. (2009) Development and evaluation of the short version of the Patient Assessment of Chronic Illness Care instrument. *Chronic Illn* **5**, 268-276, doi:10.1177/1742395309348072.
