# Supplementary material for: Depressive primary care patients’ assessment of received collaborative care
Source: Sci Rep. 2023 Feb 9;13:2329. doi: 10.1038/s41598-023-29339-9 (PMC9911390; doi:10.1038/s41598-023-29339-9)
Supplement: Supplementary file 2 — Supplementary Information 2. [file 41598_2023_29339_MOESM2_ESM.docx]

**Supplementary Material**

**Supplementary Table S1**: Practice characteristics according to included practices

|  | **pooled sample** | **PARADIES** | **PRoMPT** | **p-Value** |
| --- | --- | --- | --- | --- |
| Type of practice | N =53 | N = 31 | N = 22 |  |
| single | 34 (64.2%) | 19 (61.3%) | 15 (68.2%) | 0.822 |
| group | 19 (35.8%) | 12 (38.7%) | 7 (31.8%) |  |
| Loation of practice | 0 (0.0%) | 0 (0.0%) | 0 (0.0%) |  |
| urban | 28 (52.8%) | 8 (25.8%) | 20 (90.9%) | <0.001 |
| rural | 25 (47.2%) | 23 (74.2%) | 2 (9.1%) |  |
| Study physician qualified for "basic psychosomatic care" | 26 (49.1%) | 24 (77.4%) | 2 (9.1%) |  |
| practice founded after study specific median | 26 (51.0%) | 14 (48.3%) | 12 (54.5%) | 0.872 |

Categorical variables are presented as counts and percentages with p-values from Χ^2^-Test.

**Supplementary Text S2:** Recalibration of the PACIC 20 item version and the PACIC 11 item version.

**PARADIES**

Over the past 6 months, when I received care for my chronic conditions, what percentage of the time was I … (question)

|  | **None** |  |  |  |  |  |  |  |  |  | **Always** |
| --- | --- | --- | --- | --- | --- | --- | --- | --- | --- | --- | --- |
| **coded as** | **0** | **10** | **20** | **30** | **40** | **50** | **60** | **70** | **80** | **90** | **100** |
| Question 1 |  |  |  |  |  |  |  | x |  |  |  |
| Question 2 |  |  |  |  |  |  | x |  |  |  |  |
| Question 3 |  |  |  |  |  |  | x |  |  |  |  |
| Question 4 |  |  |  |  |  |  |  | x |  |  |  |
| Question 5 |  |  |  |  |  |  |  |  | x |  |  |
| Question 6 |  |  |  |  |  |  |  |  | x |  |  |
| Question 7 |  |  |  |  |  | x |  |  |  |  |  |
| Question 8 |  |  |  |  |  |  | x |  |  |  |  |
| Question 9 |  |  |  |  |  |  |  | x |  |  |  |
| Question 10 |  |  |  |  |  |  |  |  |  | x |  |
| Question 11 |  |  |  |  |  |  |  |  | x |  |  |

| Maximal value | 11 x 100 =1100 |
| --- | --- |
| Value in the example | 70 + 60 + 60 + 70 + 80 + 80 + 50 + 60 + 70 + 90 + 80 = 770 |
| Final value: % of Maximum | 770/1100 x 100 = **70** |

**PRoMPT**

(11 items out of the 20 item version) Over the past 6 months, when I received care for my chronic conditions, … (question)

|  | **None of the time** | **A little of the time** | **Some of the time** | **Most of the time** | **Always** |
| --- | --- | --- | --- | --- | --- |
| **coded as** | **1** | **2** | **3** | **4** | **5** |
| Question 1 |  |  | x |  |  |
| Question 2 |  |  |  | x |  |
| Question 3 |  |  |  | x |  |
| Question 4 |  |  | x |  |  |
| Question 5 |  |  | x |  |  |
| Question 6 |  |  |  |  | x |
| Question 7 |  |  |  | x |  |
| Question 8 |  |  | x |  |  |
| Question 9 |  |  |  |  | x |
| Question 10 |  |  |  | x |  |
| Question 11 |  |  |  | x |  |

| Maximal value | 11 x 5 = 55 |
| --- | --- |
| Value in the example | 3 + 4 + 4 + 3 + 3 + 5 + 4 + 3 + 5 + 4 + 4 = 42 |
| Final value: % of Maximum | 42/55 x 100 = **76.4** |
